# Vaccines, Bias, and the Perils of Non-peer-reviewed Studies: Fueling Misinformation and Vaccine Hesitancy

**DOI:** 10.1093/cid/ciag403

**Published:** 2026-08-05

**Authors:** Daniel Munblit, Danilo Buonsenso, Piero L Olliaro, Malcolm G Semple, Matthew J Greenhawt, Jon Genuneit, Liat Ashkenazi-Hoffnung, Annelies Wilder-Smith, John O Warner, Aileen Y Chang, Faisal Sultan, Nina Dwi Putri, Gregory A Poland, Andrew Bush, Viviane Boaventura, Christian Apfelbacher, Rolando Ulloa-Gutierrez, Hugh R Watson, Carlos R Oliveira, Luis Felipe Reyes

**Affiliations:** Care for Long Term Conditions Division, Florence Nightingale Faculty of Nursing, Midwifery and Palliative Care, King's College London, London, United Kingdom; Department of Paediatrics and Paediatric Infectious Diseases, Institute of Child's Health, Sechenov First Moscow State Medical University (Sechenov University), Moscow, Russia; Department of Woman and Child Health and Public Health, Fondazione Policlinico Universitario A. Gemelli IRCCS, Rome, Italy; Area Pediatrica, Dipartimento di Scienza Della Vita E Sanità Pubblica, Università Cattolica Del Sacro Cuore, Roma, Italy; The International Severe Acute Respiratory and Emerging Infection Consortium (ISARIC), Pandemic Sciences Institute, University of Oxford, Oxford, United Kingdom; NIHR Health Protection Research Unit in Emerging and Zoonotic Infections, Institute of Infection, Veterinary and Ecological Sciences, Faculty of Health and Life Sciences, University of Liverpool, Liverpool, United Kingdom; The Pandemic Institute, Liverpool, United Kingdom; Medical Scientific Council, Asthma and Allergy Foundation of America, Arlington, Virginia, USA; Institute of Public Health, Medical Faculty, Goethe-University Frankfurt, Frankfurt/Main, Germany; Department of Day Hospitalization, Schneider Children's Medical Center, Petach-Tikva, Israel; Immunization and Vaccines Department, World Health Organization, Geneva, Switzerland; Inflammation, Repair and Development Section, National Heart and Lung Institute, Faculty of Medicine, Imperial College London, London, United Kingdom; Department of Medicine, George Washington University, Washington, District of Columbia, USA; Department of Internal Medicine, Shaukat Khanum Memorial Cancer Hospital and Research Center, Lahore, Punjab, Pakistan; Department of Child Health, Cipto Mangunkusumo Hospital, Faculty of Medicine, Universitas Indonesia, Jakarta, Indonesia; Vaccine Research Group, Mayo Clinic, Rochester, Minnesota, USA; Atria Academy of Science and Medicine, New York, New York, USA; Inflammation, Repair and Development Section, National Heart and Lung Institute, Faculty of Medicine, Imperial College London, London, United Kingdom; Medicine and Precision Public Health Laboratory (MeSP²), Gonçalo Moniz Institute, Oswaldo Cruz Foundation (Fiocruz), Salvador, Bahia, Brazil; Institute of Social Medicine and Health Systems Research, Otto von Guericke University Magdeburg, Magdeburg, Germany; Pediatric Infectious Diseases Service, Hospital Nacional de Niños “Dr. Carlos Sáenz Herrera”, Caja Costarricense de Seguro Social, and School of Medicine, Universidad de Ciencias Médicas (UCIMED), San José, Costa Rica; Quayside Research, Lyon, France; Department of Clinical Medicine, Aarhus University, Aarhus, Denmark; Yale School of Medicine and Yale School of Public Health, New Haven, Connecticut, USA; The International Severe Acute Respiratory and Emerging Infection Consortium (ISARIC), Pandemic Sciences Institute, University of Oxford, Oxford, United Kingdom; Unisabana Center for Translational Science, School of Medicine, Universidad de La Sabana, Chía, Colombia

**Keywords:** vaccines, vaccine safety, vaccine hesitancy, misinformation, causal inference

## Abstract

Unvetted scientific claims about vaccines can spread rapidly and undermine decades of progress in immunization and public trust. A recent non-peer-reviewed analysis, promoted at political hearings, in the documentary “An Inconvenient Study,” and on social media, has been used to suggest that vaccinated children experience higher rates of chronic illness than unvaccinated peers. Despite standard regression models, critical flaws - uncontrolled confounding, exposure misclassification, unequal follow-up, and outcome multiplicity, invalidate causal inference. An independent group of international experts critically appraises this analysis and contrast its claims with the consistent evidence confirming vaccine safety and effectiveness. We also show how poorly designed studies can distort public discourse, amplify misinformation, and fuel vaccine hesitancy. We outline actions to strengthen research integrity, such as transparent data sharing, labeling of non-peer-reviewed content, and evidence-based communication. Safeguarding public health demands that vaccine evidence be methodologically sound, reviewed, and interpreted by qualified experts.

## Teaser

Methodologically flawed, non-peer-reviewed vaccine studies can be amplified into misinformation. Using the Lamerato analysis as a case study, this Viewpoint explains why the reported associations are not credible, summarizes robust vaccine-safety evidence, and proposes practical steps to protect public trust.

Vaccination is among the most effective public-health interventions: model-based estimates suggest that routine programs have averted 154 million deaths globally since 1974, particularly among children under 5, with health and societal benefits expected over the coming decades [1]. Program success depends not only on vaccine efficacy but also on uptake, which is driven by sustained public trust, confidence, and equitable access.

Despite these achievements, vaccine hesitancy—the delay or refusal of vaccination despite availability—has emerged as a major global health threat [2]. The phenomenon was catalyzed by the retracted 1998 Lancet paper by Andrew Wakefield et al, which falsely linked the measles, mumps, and rubella (MMR) vaccine to autism [3]. The decline in vaccination coverage led to resurgences of measles and other preventable infections, contributing to avoidable morbidity and mortality worldwide [4, 5]. Although discredited and withdrawn, the Wakefield study's influence persists through online misinformation and eroded trust in scientific institutions [6, 7].

A quarter of a century later, history is being repeated. A non-peer-reviewed analysis of a United States birth cohort, popularized by the documentary “An Inconvenient Study” (https://www.aninconvenientstudy.com) and widely circulated on social media, claims that vaccinated children have substantially higher rates of chronic illness. Despite substantial methodological flaws, the report has attracted considerable attention within anti-vaccine networks and across social media, where it is frequently cited as evidence of vaccine harm. Its rapid digital diffusion before scientific appraisal illustrates how weak analyses distort discourse and undermine confidence in immunization, particularly when amplified in political hearings [8].

We present a critical appraisal of this study by international experts in vaccinology, pediatrics, infectious diseases, epidemiology, and research methodology. We identify methodological limitations, clarify what can and cannot be inferred, and discuss implications for clinical practice, policy, and public trust.

## THE STUDY AND ITS AMPLIFICATION

The manuscript at the center of the controversy is the non-peer-reviewed analysis by Lamerato et al from a single US health system [9]. It included 18 468 children born between 2000 and 2016 who were enrolled in an integrated health plan for more than 60 days and designated the Henry Ford Health System as their primary care system. Follow-up ran from birth until plan disenrollment or 31 December 2017. Vaccination status was assembled from electronic health records and the state immunization registry, and the main analyses contrasted children who had received any vaccine with those who had received none. Outcomes were derived from diagnostic codes and grouped into a broad “chronic health condition” composite encompassing asthma, allergic and autoimmune diseases, neurodevelopmental and mental health disorders, diabetes, and malignancy. The authors report an ∼2.5-fold higher hazard of this composite outcome among vaccinated compared with unvaccinated children, with similarly increased estimates for several component conditions.

The central design problem is that the study does not define a clear, common time zero at which eligible children are classified into exposure groups and then followed under comparable ascertainment. Instead, children contribute follow-up from birth while vaccination status changes over time, yet the principal analyses are presented as if exposure were fixed. In target-trial terms, eligibility, exposure assignment, and start of follow-up are not aligned.

The document was not published in a scientific journal but appeared on the US Senate Homeland Security and Governmental Affairs website as a hearing submission and was subsequently promoted through a documentary film and widely cited by anti-vaccine influencers on social media. In response, Henry Ford Health issued a detailed fact-check cautioning that the findings are preliminary, unreviewed, and highly prone to misinterpretation [10].

Similar episodes emerged during the COVID-19 pandemic: unvetted ivermectin analyses and flawed seroprevalence reports briefly shaped policy before retraction [11]. Such examples show that the core problem is not peer review per se, but failure to meet essential standards of validity and reproducibility that underpin trustworthy research [12].

## METHODS FIRST—FIVE CRITICAL VALIDITY LIMITATIONS

The authors refer to their work as a birth cohort, birth cohort study, and retrospective study. The study relied entirely on existing administrative data, making it best understood as a historical cohort study assembled from routinely collected records. That description matters because causal interpretation in such studies depends critically on how eligibility, time zero, exposure assignment, and follow-up are specified. In this analysis, vaccination and outcomes were extracted from records created over time, but the analytic framework does not make those temporal relationships explicit.

The credibility of any observational study depends on the adequacy of its design, data sources, and analytical framework. In the case of the Lamerato et al study, several key design flaws undermine its validity (Table 1). These issues include uncontrolled confounding, surveillance bias, misclassification of exposure, unequal follow-up, and outcome heterogeneity with multiplicity, which represent fundamental departures from epidemiological standards for causal inference. While the authors acknowledge limitations to their analyses, this does not suffice to interpret their findings as robust.

**Table 1.** What a Credible Vaccine-safety Birth Cohort Study Must Do—and What the Reviewed Analysis Did Not

Design Element	Best Practice For Valid Inference	Approach in the Lamerato et al Non-Peer-Reviewed Study	Implication for Validity
Study design	Prospective or clearly time-anchored cohort with defined enrollment at or near birth and systematic follow-up using uniform criteria	The authors described the analysis as a birth cohort and retrospective. It relied on administrative data for children born between 2000 and 2016; follow-up length differed by vaccination status	Temporal bias and incomplete ascertainment of later-onset outcomes
Exposure definition	Detailed vaccination data (type, dose, timing) modeled as a time-varying covariate	Binary classification (“any vaccination” vs “none”) treated as fixed	Time-related misclassification; classical immortal-time bias would usually favor vaccination
Confounding control	Adjustment for socioeconomic, behavioral, environmental, and healthcare-use variables within a causal framework (eg DAGs)	Adjustment limited to perinatal factors (sex, race, birthweight, prematurity)	Profound residual confounding; nonexchangeable comparison groups
Healthcare contact	Equalized or modeled encounter frequency and use of active comparators	Vaccinated children had far more clinical encounters (≈7 vs 2 per year)	Surveillance and diagnostic opportunity bias inflate recorded diagnosis rates
Outcome definition	Validated, homogeneous endpoints and limited number of prespecified outcomes	Heterogeneous composite of unrelated chronic conditions; dozens of exploratory outcomes	Multiplicity, statistical noise, outcome heterogeneity
Follow-up duration	Similar observation windows; censoring handled appropriately	Median follow-up is roughly twice as long for vaccinated children	Differential opportunity for diagnosis
Analytical transparency	Preregistered protocol; open code and data	None reported; no public protocol	Post hoc analyses and selective reporting are likely
Peer review	Independent scientific evaluation before dissemination	Uploaded to the US Senate website and not peer-reviewed	High potential for error and misinterpretation

### Unmeasured and Residual Confounding

Vaccination status is nonrandomly distributed and associated with socioeconomic, demographic, behavioral, and healthcare factors [13, 14]. Parents who decline vaccination systematically differ in education, income, health beliefs, and healthcare-seeking behavior—factors influencing virtually every chronic outcome in this analysis. Although race was included as an adjustment variable, race and ethnicity recorded in administrative systems are often incomplete or coarse and cannot substitute for direct measurement of the broader social determinants that shape both vaccination and subsequent diagnosis.

The analysis adjusted only for birth-related covariates (sex, race, birthweight, prematurity, respiratory distress, and birth trauma); no adjustment was made for parental socioeconomic status, education, occupation, healthcare-seeking behavior, or childcare attendance. The observed associations are far more plausibly explained by population differences than vaccination.

Established frameworks for evaluating exposure studies, such as ROBINS-E and JBI guidance for etiology reviews, identify uncontrolled confounding as a major threat to validity. In this case, nearly every social, environmental, and behavioral determinant of health is associated with vaccination status, producing nonexchangeable comparison groups. As illustrated in Table 1 and Figure 1, factors such as socioeconomic status, environment, healthcare-seeking behavior, and prematurity influence both vaccination and diagnosis of chronic disease and open multiple backdoor paths capable of generating spurious associations.

**Figure 1.**
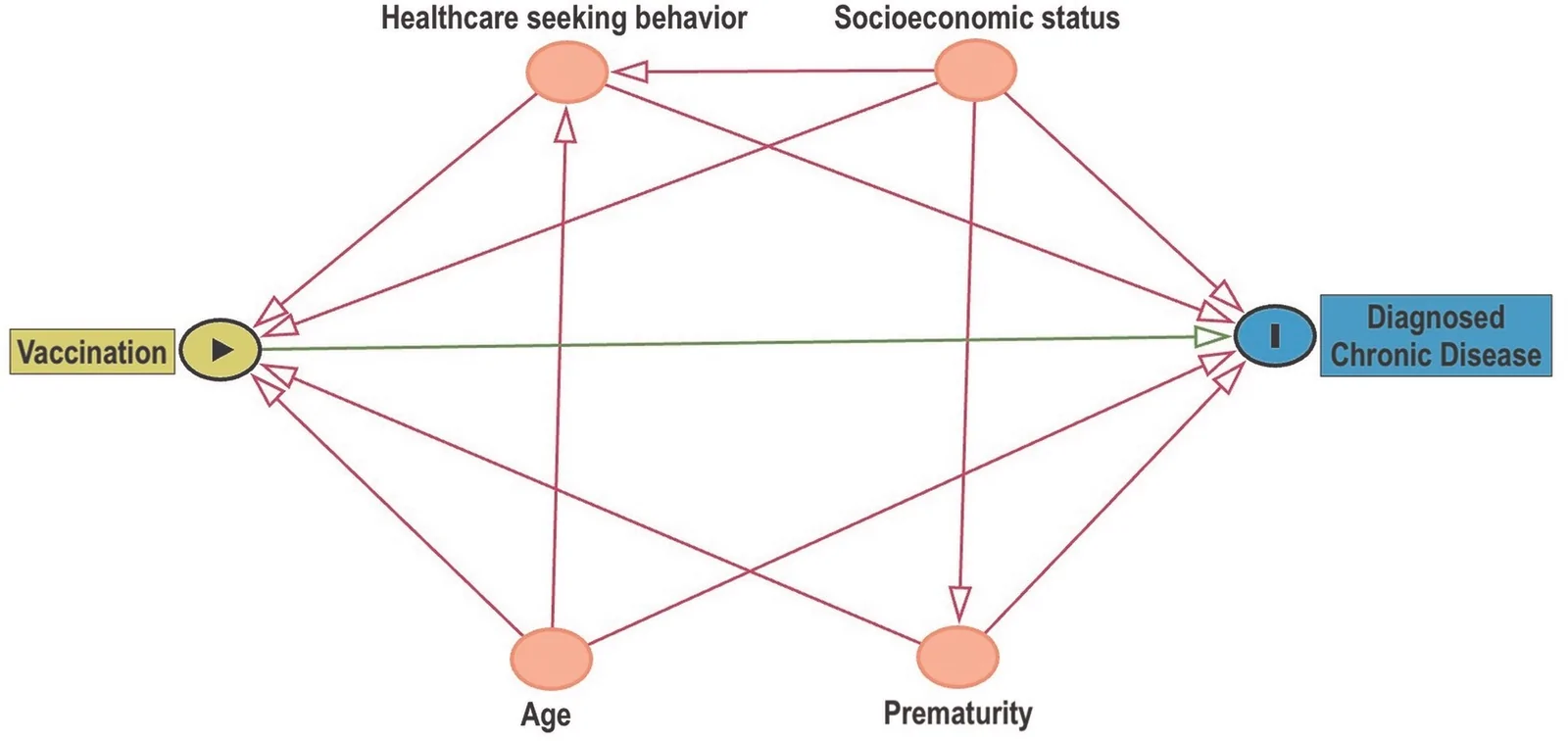
Directed acyclic graph (DAG) illustrating the relationship between vaccination and diagnosed chronic dis ease. Arrows represent assumed causal pathways. Socioeconomic status, age, prematurity, and healthcare-seeking behaviour ar e potential confounders that influence both vaccination and the likelihood of diagnosis. This model illustrates diagnostic opportu nity bias: individuals with greater healthcare engagement are more likely to be vaccinated and to have a diagnosis recorded. Co nditioning on enrolment and captured healthcare contact may also induce selection (collider) bias. When this bias is not adequat ely controlled, it can create an apparent association between vaccination and disease diagnoses even when true disease rates ar e equal.

### Surveillance, Ascertainment Bias, and Missingness

Surveillance (ascertainment) bias, sometimes termed informed presence bias when healthcare use itself affects recorded diagnoses, is particularly problematic when exposure correlates with healthcare utilization, as in vaccination programs requiring repeated clinical visits. Vaccinated children had ∼7 vs ∼2 visits/year for unvaccinated children, creating substantial differential opportunity for detection. Many pediatric conditions, such as otitis media, eczema, mild asthma, and speech delay, are clinically ascertained rather than self-reported. They are detected only in children who present for healthcare. Infrequent attenders, therefore, appear “artificially” healthy.

While the authors attempt to control for this by restricting to individuals with at least one encounter, this does not correct for intensity, frequency, or continuity of care. Without modeling the frequency of healthcare contact as a time-dependent covariate or using designs that balance encounter opportunities (eg matched active comparators), the direction of bias is predictable. The pattern of higher risks for common, mild conditions but no increase, or even a decrease, for rare, severe diseases (such as cancer) suggests the results reflect differences in diagnostic opportunity rather than actual biological effects.

Missing data further compounds these biases. Children with fewer healthcare visits may have exited the health system, resulting in missing vaccination records or undiagnosed conditions, particularly if care occurred outside the surveillance network. This leads to differential misclassification and outcome under-ascertainment in the unvaccinated group. Because missingness is tied to both exposure and outcome, standard adjustments are insufficient. In addition, conditioning analyses on continued enrollment or healthcare contact can introduce selection or collider bias when those factors are related to both vaccination behavior and underlying health. Without transparent reporting on missing data and its handling, the risk of systematic, directional bias may be high.

### Misclassification and Time-varying Exposure

The report describes vaccination as a binary exposure (“any vaccine” vs “none”) and classifies outcomes according to whether vaccination occurred before the first recorded diagnosis. However, the statistical models presented treat vaccination status as fixed at baseline, without documentation of how time-varying exposure was handled.

In longitudinal data, children initially unvaccinated contribute person-time before receiving their first vaccine. This means vaccination should be analyzed as a time-varying exposure. A target-trial or trial-emulation framework would align eligibility, time zero, exposure assignment, and follow-up, and could be implemented using time-dependent Cox models or related methods such as cloned or sequential trial approaches [15]. Classical immortal-time bias, misallocating guaranteed event-free time to the vaccinated group, would usually make vaccination appear spuriously protective, not harmful. The broader problem here is that the paper does not show how changing exposure status over time was handled, leaving substantial scope for time-related misclassification and distorted hazard ratios.

The exposure definition further collapses all vaccines, doses, and timing into a single dichotomous category, disregarding variation in schedule adherence, vaccine type, and sequence. Such simplification precludes meaningful assessment of dose–response relationships or causal pathways and prevents valid temporal interpretation of the observed associations.

### Unequal Follow-up and Age-dependent Outcomes

Median follow-up was 970 days for vaccinated and 461 days for unvaccinated children, approximately a 2-fold difference. As the total number of vaccinated participants was substantially larger, the difference in cumulative person-time of observation may be even greater, further amplifying the imbalance between groups. Many outcomes, most notably neurodevelopmental disorders, asthma, autoimmune disease, and malignancy, manifest after the first few years of life. Shorter observation among unvaccinated participants virtually guarantees under-ascertainment of these later outcomes, artificially lowering event counts for unvaccinated children and inflating relative hazards for vaccinated children.

The authors present Kaplan–Meier disease-free survival curves without addressing the censoring mechanism. Disenrollment terminates observation and is related to both vaccination and healthcare engagement, so censoring is unlikely to be noninformative, biasing the survival comparison directly. Competing risks are a minor concern given only 6 deaths; the issue is informative censoring, not a competing event. No assessment of the proportional-hazards assumption is reported. Although the published curves show no clear crossing that would by itself invalidate proportional hazards, the absence of any reported diagnostic assessment is a further indicator of limited analytical transparency. No attempt is made to equalize observation windows through age-matching, left-truncation, or incidence-rate modeling. Standard practice in vaccine safety and effectiveness studies, such as anchoring analyses to define risk periods or using age as the time scale, is absent. This temporal imbalance interacts with the exposure misclassification described above, further biasing results.

### Outcome Heterogeneity and Multiplicity

Another significant limitation of the analysis concerns the extensive multiplicity of comparisons. The authors examined a large composite labeled “chronic health condition” together with numerous individual outcomes and subgroup analyses yet applied no statistical adjustment for multiple testing. When many outcomes are tested simultaneously, the expected number of false-positive associations rises sharply, even under the null hypothesis of no true effect, several nominally significant findings would arise by chance. The dataset's structure amplifies the magnitude of this problem as some outcomes are rare, others common, and the number of events in the unvaccinated group is often minimal. As a result, several hazard ratios are derived from unstable estimates with wide confidence intervals or zero-event cells yet are presented as meaningful differences. The analysis, therefore, cannot distinguish genuine signals from statistical noise.

### Cumulative Impact

Limited confounder control, differential healthcare contact, crude exposure classification, truncated follow-up, and outcome multiplicity could each substantially distort effect estimates; together, they could yield hazard ratios of the magnitude reported. The direction of these biases is mainly predictable—children who are more engaged with the healthcare system over a longer period (and therefore more likely to be vaccinated) also accumulate more coded diagnoses.

Taken together, the limitations described severely compromise the internal validity of the analysis. Established frameworks for evaluating nonrandomized exposure studies, such as ROBINS-E, and for grading overall certainty of evidence, would classify the study as being at critical risk of bias and the certainty of the evidence as very low. The findings should therefore be interpreted not as evidence of vaccine-related harm, but as an illustration of how insufficient conduct and analysis of epidemiological research, combined with post hoc analysis and public amplification outside peer review, can generate spurious associations that appear convincing. Plain-language explanations of key methodological terms are provided in Box 1 to support interpretability for non-specialist readers.

Box 1. **Plain-language Explanations of Key Methodological Concepts Discussed in This Viewpoint**

**Table:** 

Methodological Term	Plain-language Explanation	Example
Confounding (including unmeasured/residual confounding)	Confounding happens when vaccinated and unvaccinated children differ in important ways that also affect their chance of later illness. If these differences are not fully measured and adjusted for, they can create the illusion that vaccines cause harm (or benefit) when the real cause is something else.	If unvaccinated children are more likely to have parents who avoid doctors and decline screening, they may have fewer recorded diagnoses of chronic disease, even if their true underlying health is no better, and this can make vaccines look falsely harmful.
Surveillance/ascertainment bias	Surveillance (or ascertainment) bias means that people who see doctors more often are more likely to be diagnosed with conditions, so they look “sicker” in the data even if their true health is similar to those who rarely attend.	If vaccinated children are brought to clinics more often for routine well-child visits, they will naturally have more chances to have problems detected and coded in the record. Unvaccinated children who rarely attend may have fewer recorded diagnoses, not because they are healthier, but because no one is looking.
Diagnostic opportunity bias (a specific form of surveillance bias)	Diagnostic opportunity bias is the idea that more healthcare contact means more opportunities to find and record disease. It is not that vaccination itself causes disease, but that vaccinated children are seen more often and therefore have more chances to receive a diagnosis.	A child who sees a doctor 10 times a year has 10 chances to have asthma or ADHD recognized and coded. A child who sees a doctor once has only 1 chance. If vaccinated children are in the first group and unvaccinated in the second, the data may misleadingly suggest that vaccination “causes” these conditions.
Misclassification of exposure (and time-varying exposure)	Misclassification occurs when children are put into the wrong vaccination category in the analysis. Treating vaccination as if it were fixed at the start of follow-up, when in reality children move from unvaccinated to partly vaccinated to fully vaccinated over time, can seriously distort results.	If a child is counted as “vaccinated” for the entire follow-up period, statistical models will wrongly credit the “vaccinated” group with illness events that actually occurred before the child received any vaccines. This can make vaccines look riskier than they are.
Immortal-time bias	Immortal-time bias refers to a period during follow-up when participants must remain alive and event-free to qualify for a later exposure category. If that guaranteed event-free time is wrongly allocated to the exposed group, it usually makes the exposure look safer than it really is.	To be classified as “fully vaccinated,” a child must (by definition) survive and remain event-free long enough to receive all scheduled doses. If the analysis counts this lead-in period as time “in the vaccinated group,” it can give that group an artificial advantage because children who became ill or died earlier could never enter that category.
Multiple testing/multiplicity	Multiplicity means running many statistical tests across many outcomes, time windows, and subgroups. Even when vaccines have no harmful effect, some results will look “statistically significant” purely by chance.	If you look at 20 different outcomes and use a 1-in-20 (5%) significance threshold, you would expect about one “significant” result just by chance, even if vaccines have no true effect on any outcome. When hundreds of comparisons are made, a few large or “significant” risk estimates are almost guaranteed to appear, and these can easily be overinterpreted.
Selection/collider bias	Selection bias can arise when entering the study, staying under observation, or having data recorded depends on factors related to both vaccination and illness. Conditioning analyses on such factors can create a misleading link between vaccination and disease even when none exists.	If children had to stay enrolled in a particular health plan and keep using the same health system to be included, families who vaccinate regularly and families who seek care more often may be disproportionately retained. Comparing those selected groups can generate an association between vaccination and diagnosis that reflects how the sample was assembled, not a true biological effect.

### What Robust Vaccine-safety Evidence Already Shows

The conclusions of this non-peer-reviewed study stand in contrast to biologic plausibility and the extensive body of peer-reviewed evidence accumulated over several decades. Vaccine safety has been investigated through a continuum of complementary approaches, including randomized trials, post-marketing pharmacovigilance, population-based cohort studies, and large record-linkage systems, all being subject to independent oversight and reproducibility standards [16]. Their findings are consistent: serious adverse events following immunization are rare, typically transient, and are vastly outweighed by the benefits of disease prevention. Anaphylaxis is one well-recognized example, occurring on the order of 1–2 cases per million doses administered [17]. Other authentic safety signals, when they arise, are generally specific, biologically plausible, and detectable through post-marketing surveillance systems.

Rigorous studies have addressed specific public concerns. A Danish nationwide cohort (n = 657 461; 2019) found no increased autism risk with MMR (aHR 0.93, 95% CI .85–1.02) [18]. Consistent null findings across multiple designs and decades have effectively resolved this question [19, 20].

Other vaccines have been examined with equal scrutiny. Extensive observational analyses show no credible link between routine childhood vaccination and chronic disease [21], including asthma, allergic disease [22], autoimmune conditions, neurodevelopmental disorders, or malignancy. Where genuine safety signals have emerged, such as myocarditis following mRNA COVID-19 vaccination or thrombosis with adenoviral-vector vaccines, they have been rapidly identified, quantified, and incorporated into updated policy guidance [16]. These examples illustrate that the global vaccine-safety infrastructure is both sensitive to real risks and responsive to new evidence.

Decades of varied research support a clear conclusion: routine childhood immunization does not increase chronic disease risk. Under-vaccination harms remain well-documented and pose immediate health threats [4]. Given this concordant evidence, further “vaccinated vs unvaccinated” cohort studies are unlikely to change this conclusion. Future research should instead focus on specific, biologically plausible safety questions, and on improving causal inference in observational vaccine studies more broadly [23]. A credible study addressing chronic disease and vaccination would require clearly defined enrollment at or near birth, balanced follow-up periods, detailed time-varying vaccination data, extensive adjustment for socioeconomic and healthcare-use variables, explicit handling of confounding and misclassification biases [24], and transparent, preregistered analysis plans (Table 1). Without these elements, new analyses risk repeating the same methodological errors rather than contributing meaningfully to science or public reassurance [8].

## PUBLIC-HEALTH CONSEQUENCES OF MISINFORMATION AND RECOMMENDATIONS FOR RESPONSE

The rapid spread of unvetted scientific claims has real and measurable public-health consequences. Studies consistently show that exposure to vaccine misinformation can reduce vaccination intent and trust in health authorities even after correction [25]. During the COVID-19 pandemic, misinformation on social media and alternative platforms was linked to declines in vaccine uptake, echoing the earlier MMR–autism controversy, where the retracted 1998 Lancet paper [3] triggered sustained drops in measles coverage and subsequent outbreaks.

Episodes such as the promotion of “An Inconvenient Study” follow a familiar pattern—a complex scientific question is simplified into a provocative narrative, bypassing peer review and amplified through political hearings, documentaries, and influencers. Once circulated through algorithmic platforms, emotionally charged messages invoking harm to children spread faster than any correction, creating a persistent asymmetry of misinformation and harm. The same ecosystem increasingly includes AI-generated and AI-amplified content, which may further magnify misleading medical narratives when large language models prioritize apparent helpfulness or agreement over accuracy [26]. The recent “bixonimania” experiment demonstrated this vulnerability directly—a researcher uploaded 2 deliberately fabricated preprints describing a fictitious medical condition to a preprint server, and within weeks major AI chatbots were presenting the invented illness as a legitimate diagnosis to users seeking health information [27]. The fake papers were subsequently cited in peer-reviewed literature, suggesting that AI-mediated misinformation can infiltrate not only public discourse but also the scientific record itself.

Countering misinformation requires coordinated action across research, clinical practice, and media (Box 2). Researchers should preregister analyses and avoid speculative interpretation. The problem is not preprints per se, but uncontextualized amplification of unreviewed claims as established evidence. The claim made at the Senate hearing that the decision not to submit the study for publication reflected fear of professional repercussions is unconvincing [8] and reflects a substantial lack of understanding of the scientific process; research integrity is best preserved through transparent scrutiny, not through amplification in social-media echo chambers. Clinicians should frame conversations around vaccine evidence while acknowledging legitimate safety questions. Parents should discuss concerns with trusted providers rather than social media. Re-engagement must be 2-way: listening to concerns, acknowledging historical mistrust, and working with trusted local messengers is more effective than one-directional correction. In an environment where the independence and composition of advisory bodies may themselves be contested, the credibility of vaccine guidance rests less on institutional authority alone and more on the transparency and diversity of the expertise underpinning it—including vaccinology, pediatric and adult medicine, epidemiology, biostatistics, and public health.

Box 2. **Recommendations to Address Misinformation and Uphold Research Integrity**

**Table:** 

Stakeholder	Recommendations
Researchers	Protocols and analysis plans are publicly preregistered before data collection. Analytic code and deidentified data are shared transparently, under governance frameworks that prevent selective disclosure. Associations are clearly distinguished from causation in publications and media communication. Independent peer review precedes public release, with policymakers briefed in advance on main findings and limitations.
Journals and preprint servers	Non-peer-reviewed work is clearly labeled, with contextual expert commentary for studies influencing public opinion or policy. Rapid expert review provides commentary or provisional appraisal of widely circulated manuscripts within 48–72 h. Open data, reporting standards, and transparent disclosures are enforced, with visible indexing of retractions and corrections.
Clinicians and professional societies	Clinicians and professional societies engage the public with empathy and evidence, explaining the evolving nature of science. Vaccination benefits and safety are communicated clearly, with uncertainties addressed openly. Communication hubs deliver unified, evidence-based messages quickly during misinformation events.
Public-health agencies and communicators	Clear, evidence-based communication conveys complex science accessibly and builds trust through openness about uncertainty. Partnerships with communicators and media are strengthened to extend reliable information and address emerging misinformation. Public-health initiatives integrate education on data literacy, scientific reasoning, and consensus-building. Experts in risk and scientific communication are routinely engaged to co-design and test messages before wide dissemination.
Policymakers and media	When non-peer-reviewed claims are discussed in public or policy settings, they should be clearly labeled as preliminary and accompanied by independent expert appraisal of their methodological limitations. Independent scientific-advisory mechanisms appraise primary research that informs major policy decisions. Support for scientific literacy, transparency, and misinformation resilience is prioritized.
Social-media influencers and digital communicators	Scientific claims are verified against peer-reviewed or authoritative sources before sharing. All sponsorships and partnerships are transparently disclosed, with affiliations clearly stated. Engagement metrics are balanced with accuracy, prioritizing clarity and reliability over virality. Collaboration with qualified experts is sought when interpreting or presenting health-related research instead of self-interpretation.

Factual correction alone is insufficient. Evidence shows that prebunking, timely fact-checking, and trusted communicators reduce misinformation's influence on social media [28]. Public-health agencies should produce accessible, shareable content that conveys scientific accuracy with comparable emotional resonance. Equally important, the public requires education in data literacy and statistical interpretation.

Public institutions, policymakers, and media outlets should not present unpublished analyses as established evidence. When non-peer-reviewed claims are discussed in public or policy settings, they should be clearly labeled as preliminary and accompanied by independent expert appraisal of methodological limitations. This approach does not require regulatory censorship; it promotes labeling of non-peer-reviewed work, linkage of preprints to later peer-reviewed publication or retraction, and independent appraisal before amplification. Evidence informing health decisions must be interpreted by people with appropriate methodological and clinical training and must undergo transparent review before public dissemination. When politicians replace expert evaluation with advocacy, they distort public understanding, erode trust, and may harm health outcomes. Upholding rigorous evidentiary standards is a shared responsibility, safeguarding scientific integrity and public health.
